# Early severe impairment of hematopoietic stem and progenitor cells from the bone marrow caused by CLP sepsis and endotoxemia in a humanized mice model

**DOI:** 10.1186/s13287-015-0135-9

**Published:** 2015-08-14

**Authors:** Tomasz Skirecki, Jerzy Kawiak, Eugeniusz Machaj, Zygmunt Pojda, Danuta Wasilewska, Jarosław Czubak, Grażyna Hoser

**Affiliations:** Department of Flow Cytometry, The Center of Postgraduate Medical Education, Marymoncka 99/103, 01-813 Warsaw, Poland; Department of Anesthesiology and Intensive Care Medicine, The Center of Postgraduate Medical Education, Czerniakowska 231, 00-416 Warsaw, Poland; Department of Cellular Engineering, Maria Sklodowska-Curie Memorial Cancer Center and Institute of Oncology, W. Roentgena 5, 02-781 Warsaw, Poland; Department of Clinical Cytology, The Center of Postgraduate Medical Education, Marymocnka 99/103, 01-813 Warsaw, Poland; Department of Pediatric Orthopedics, The Center of Postgraduate Medical Education, Konarskiego 13, 05-400 Otwock, Poland

## Abstract

**Introduction:**

An effective immune response to severe bacterial infections requires a robust production of the innate immunity cells from hematopoietic stem and progenitor cells (HSPCs) in a process called emergency myelopoiesis. In sepsis, an altered immune response that leads to a failure of bacterial clearance is often observed. In this study, we aimed to evaluate the impact of sepsis on human HSPCs in the bone marrow (BM) microenvironment of humanized mice subjected to acute endotoxemia and polymicrobial sepsis.

**Methods:**

Humanized mice (hu-NSG) were generated by transplanting NOD.Cg-Prkdc/scidIL2rγ (NSG) mice with the human cord blood CD34^+^ cells. Eight weeks after the transplantation, hu-NSG mice were subjected to sepsis induced by endotoxemia—*Escherichia coli* lipopolysaccharide (LPS)—or by cecal ligation and puncture (CLP). Twenty-four hours later, HSPCs from BM were analyzed by flow cytometry and colony-forming unit (CFU) assay. CLP after inhibition of Notch signaling was also performed. The effects of LPS on the in vitro proliferation of CD34^+^ cells from human BM were tested by CellTrace Violet dye staining.

**Results:**

The expression of Toll-like receptor 4 receptor was present among engrafted human HSPCs. Both CLP and endotoxemia decreased (by 43 % and 37 %) cellularity of the BM. In addition, in both models, accumulation of early CD34^+^ CD38^−^ HSCs was observed, but the number of CD34^+^ CD38^+^ progenitors decreased. After CLP, there was a 1.5-fold increase of proliferating CD34^+^ CD38^−^Ki-67^+^ cells. Moreover, CFU assay revealed a depressed (by 75 % after LPS and by 50 % after CLP) production of human hematopoietic colonies from the BM of septic mice. In contrast, in vitro LPS stimulated differentiation of CD34^+^ CD38^−^ HSCs but did not induce proliferation of these cells in contrast to the CD34^+^ CD38^+^ progenitors. CLP sepsis modulated the BM microenvironment by upregulation of Jagged-1 expression on non-hematopoietic cells, and the proliferation of HSCs was Notch-dependent.

**Conclusions:**

CLP sepsis and endotoxemia induced a similar expansion and proliferation of early HSCs in the BM, while committed progenitors decreased. It is suggestive that the Notch pathway contributed to this effect. Targeting early hematopoiesis may be considered as a viable alternative in the existing arsenal of supportive therapies in sepsis.

## Introduction

Despite the continuous progress in critical care medicine and anti-microbial therapies, sepsis and septic shock remain a serious health-care problem worldwide. The morbidity due to sepsis reaches 50–95 cases per 100,000 citizens in the USA annually [1], and the average mortality rates are high: 41 % in Europe and 28 % in the USA [2]. It has been speculated in recent years that the complex pathophysiology of sepsis coupled with the highly heterogeneous makeup of patients with sepsis has hindered successful development of specific anti-sepsis drugs. Thus far, the major improvement in outcome has been achieved by introduction of the Sepsis Surviving Campaign Guidelines in 2004 [3].

The central role of immune system disturbances in sepsis pathophysiology has been well recognized, and an effort was made to comprehensively categorize those disturbances. At the cellular level, fight against an infection requires massive production of immune-competent cells of the innate immunity. This process is called emergency myelopoiesis and involves a robust proliferation of hematopoietic progenitors and progression of dormant hematopoietic stem cells (HSCs) into the cell cycle [4]. The emergency hematopoiesis represents a physiological response of the immune system to infections that is governed by (a) direct stimulation of progenitor cells via Toll-like receptors (TLRs) [5], (b) production of growth factors and cytokines by the bone marrow (BM) niche-forming cells and mature hematopoietic cells (like granulocyte colony-stimulating factor, or G-CSF) [6], and (c) paracrine effects of TLR-activated HSCs [7]. To maintain hematopoietic homeostasis, a balance between self-renewal and differentiation of ‘true’ HSCs must be maintained. It was shown in the mouse that chronic inflammatory stimulation leads to an exhaustion of HSCs in a model of multiple low-dose lipopolysaccharide (LPS) injections [8]. Also, TLR stimulation was reported to skew HSC differentiation toward myeloid lineages [5]. Given that existing studies point out that many patients with sepsis die with signs of compromised immune defense and ongoing infections [9], characterization of the emergency myelopoiesis dynamics in sepsis is highly warranted. Although patients with sepsis typically present with a robust blood leukocytosis, marked leukopenia has been frequently reported in other subjects with sepsis. In fact, both reactions are included in the diagnostic criteria of sepsis [10]. It is currently not known whether leukopenia is a consequence of the failure of HSC response or an inadequate signaling in the BM microenvironment. Yet, to date, no clinical studies have characterized distribution of the BM stem and progenitor cells and their potential modulation by sepsis syndromes. The existing data on hematopoiesis under infectious conditions come exclusively from experimental models of infections and sepsis in mice. Intriguingly, the data regarding HSCs and their progeny are conflicting. Whereas the model of cecum ligation and puncture (CLP) septic peritonitis led to an expansion of both long-term HSCs (LT-HSCs) and short-term HSCs (ST-HSCs) [11], *Pseudomonas aeruginosa* sepsis and LPS endotoxemia expanded only the LT-HSCs. However, LT-HSC functionality was compromised as shown in the repopulating experiments [12]. Similarly, an intravenous injection of heat-killed *Escherichia coli* was demonstrated to expand HSCs at the expense of myeloid progeny [13]. Although several mechanisms of the HSC expansion in those conditions have been proposed (e.g., proliferation of HSCs, block of differentiation, and ‘de-differentiation’ of committed progenitors), it remains to be evaluated whether similar processes occur in human sepsis.

The relevance of used mouse models of critical care diseases has been recently vigorously discussed [14]. The recently developed humanized mice models that rely on hu-HSC transplantation offer a high clinical relevance to multiple investigative and mechanistic approaches in those mice [15]. For example, CLP sepsis in humanized mice has already been shown to mimic some of the features of immune disturbances observed in patients with sepsis, including production of human cytokines and apoptosis of human CD3 lymphocytes [16]. After intravenous transplantation, hu-HSCs home to murine BM in an SDF-1-CXCR4-dependent manner [17] and recreate histological positioning within the bone structure similar to the one present in humans [18]. In regard to recapitulation of clinical sepsis syndromes, several animal models have been developed and used in the last decades. One of the most common approaches is an injection of bacterial LPS. Although it is currently not considered an appropriate simulation of sepsis [19], it nevertheless constitutes a simple and reproducible experimental system which evokes a strong and transient release of inflammatory mediators. In contrast, CLP has been deemed much more clinically relevant given that it reproduces many aspects of human septic peritonitis [19]. Although various endpoints were previously compared in those two models (i.e., LPS and CLP), a comparison of endotoxemia and sepsis impact upon the hematopoietic stem and progenitor cells (HSPCs) in the BM microenvironment in a humanized mouse model system has been never attempted.

In the present study, we aimed to investigate the modulatory effects of CLP sepsis and LPS endotoxemia on human HSPCs in the BM of the NOD.Cg-Prkdc/scidIL2rγ (NSG) humanized mouse model. Specifically, we set out (1) to characterize potential changes in the phenotypes or markers (or both) of proliferation of the grafted human cells and (2) to examine the proliferation capacity of the grafted human hematopoietic progenitor cells (HPCs).

## Methods

### Human umbilical cord blood CD34^+^ cells

Human umbilical cord blood (UCB) samples were processed in accordance with the procedures approved by the Institutional Review Board of the Maria Sklodowska-Curie Memorial Cancer Center and Institute of Oncology. The UCB units from healthy donor mothers were obtained with their informed consent. The UCB (30–50 ml) was drained with the standard Baxter set with citrate volume reduced to 23 ml. Numbers of white blood cells and mononuclear cells were estimated by using a Sysmex 820 semiautomatic hematological analyzer (Sysmex Co., Kobe, Japan). Next, blood diluted 1:1 with phosphate-buffered saline (PBS) was applied to Ficoll (Invitrogen Ltd, Paisley, UK)-Uropoline (Polpharma SA, Starogard Gdanski, Poland) of density of 1.077 g/ml and centrifuged for 40 min at 400 × *g* at 4 °C. Isolated cells were frozen in liquid nitrogen in Dulbecco’s modified Eagle’s medium (Invitrogen Ltd) containing 50 % inactivated fetal bovine serum (FBS) (Pan-Biotech GmbH, Aidenbach, Germany) and 10 % dimethyl sulfoxide (DMSO) (Sigma-Aldrich, St. Louis, MO, USA).

Enrichment of human HSCs was performed by isolation of CD34^+^ cells from thawed UCB mononuclear cells. CD34^+^ were separated by the positive immune separation method by using an EasySep CD34 kit (Stemcell Technologies, Vancouver, BC, Canada) in accordance with the protocol of the manufacturer. Purity of the isolated cells was evaluated by flow cytometry after staining with anti-CD45 FITC/anti-CD34 PE antibody cocktail (BD Biosciences, San Jose, CA, USA). Each isolation resulted in CD34^+^ cell purity of more than 90 %.

### Mice and transplantation of CD34^+^ cells

NSG breeding pairs were obtained from The Jackson Laboratory (Bar Harbor, ME, USA) and expanded and maintained in the animal facility of the Center of Postgraduate Medical Education (Warsaw, Poland). Mice were kept under pathogen-free conditions and fed with standard sterilized diet and sterile drinking water *ad libitum*. The animal study was approved by Local Ethics Committee No. 4 in Warsaw, Poland.

Four- to five-week-old female mice received intraperitoneal (i.p.) injection of busulfan (Sigma-Aldrich) for two consecutive days before cell transplantation. Mice were injected with the busulfan dose of 25 mg/kg in 100 μl of 30 % DMSO in saline. Twenty-four hours after the last myeloablative injection, mice received intravenous infusion (via a tail vein) of 10^5^ purified UCB CD34^+^ cells in 100 μl of 0.9 % NaCl. After the transplantation, mice were observed and bred for further experiments.

### Model of LPS endotoxemia

Because of the variability in the level of human cell chimerism after the transplantation of CD34^+^ cells, the injected mice were pair-matched before the proper experiments. Seven weeks after transplantation, 50 μl of the peripheral blood was collected from the tail vein and placed in the EDTA-containing tubes. Next, the total cell count was evaluated (by using Bürker chamber and Türk’s solution) and the percentage of human CD45^+^ cells was analyzed by flow cytometry after staining with anti-CD45 PE antibody (BD Biosciences). Animals with chimerism varying less that ±2 % were matched into pairs and subjected to experimental endotoxemia or CLP models. Eight weeks after the transplantation, the selected paired mice were subjected to endotoxemia, receiving an intravenous injection of 40 μg of LPS from *E. coli* O55:B5 (Sigma-Aldrich) in 100 μl of 0.9 % saline or 100 μl of saline as control.

### Model of CLP sepsis

A CLP model of sepsis of moderate severity was also performed 8 weeks after transplantation in accordance with the original protocol developed by Chaudry’s lab [20] with additional modifications [21]. In brief, mice were anesthetized with i.p. injection of sodium pentobarbital (Sigma-Aldrich) with a dose of 30 mg/kg. A midline incision was made, and after externalization, the cecum was ligated (1 cm from the apex) and punctured twice (through-through) with a 22-G needle. Next, a small amount of fecal mass from the punctured cecum was gently squeezed out to ensure patency of punctures, cecum was relocated, and 4/0 sutures were used to close the peritoneum and skin. Sham-operated mice underwent only incision and cecum exteriorization.

Twenty-four hours after induction of endotoxemia or CLP, mice were anesthetized with sodium pentobarbital (200 mg/kg), bled from the retro-orbital plexus into EDTA, and terminated. The BM from each femur was flushed out with 0.5 ml of PBS. From some control mice, splenocytes were isolated by mashing the spleen with a needle. Blood and BM cells were counted by using Bürker chamber and Türk’s solution.

### Primary human bone marrow CD34^+^ cells in vitro stimulation

Human BM was collected during the elective hip osteotomy from patients without severe comorbidities. Informed consent was obtained from all patients, and the study was approved by the local ethics board of the Center of Postgraduate Medical Education (Warsaw, Poland). After red blood cell lysis, CD34^+^ cells were isolated with an EasySep CD34 kit as described above. Freshly isolated BM CD34^+^ cells (4 × 10^5^) were stained with CellTrace Violet dye (Life Technologies, Carlsbad, CA, USA) in accordance with the protocol of the manufacturer. Next, cells were resuspended in StemSpam medium (Stemcell Technologies) with 10 % FBS, split, and cultured in 96-well plate with 1 μg/ml of LPS (*E. coli* O55:B5, Sigma-Aldrich) or control medium. Cells were cultured in a humidified incubator with low oxygen concentration: 1 % O_2_, 5 % CO_2_ and 94 % N_2_ for 9 days. To trace their proliferation history, cells were stained after 9 days with anti-CD34 FITC, anti-CD38 PE.Cy7, and 7-AAD (BD Biosciences) and analyzed by flow cytometry.

### Flow cytometry analysis

Immunophenotyping analysis of human cells was performed by using a flow cytometry technique. Briefly, 70 μl of cell suspension was incubated for 5 min with 5 μl of inactivated mice serum to block murine Fc receptors. Then, 5 μl of monoclonal antibodies—CD34-APC, CD34 PE.Cy7, CD38-PE.Cy7, TLR4-PE, CXCR4-biotin + streptavidin APC.Cy7, Lin-FITC (CD19, CD2, CD3, CD14, CD66b, CD24, CD16, CD56, and CD235a), CD45-AmCyan, CD19-PE, CD3-FITC, CD33-PerCP, CD14-PE, CD235a-APC, and CXCR4-biotin (BD Biosciences), TLR4-PE (R&D Systems, Minneapolis, MN, USA), and G-CSFR-APC (BioLegend, San Diego, CA, USA)—was added and incubated for 30 min at room temperature. After staining, red blood cells were lysed with BD Pharm Lyse solution (BD Biosciences) for 10 min, washed with PBS with 2 % Newborn Calf Serum (NCS), and resuspended in 0.5 % paraformaldehyde in PBS. For Ki-67 and Notch Intracellular Domain (NICD) intracellular staining, fixed cells were washed with PBS and permeabilized by 0.25 % Triton X-100 in PBS. Next, 5 μl of anti-Ki-67 PE antibody, isotype control (BD Biosciences), or anti-NICD (Cell Signaling Technology, Danvers, MA, USA) was added for 30 min, and after PBS wash, cells were resuspended again in paraformaldehyde. Prior to use, the anti-NICD antibody was labeled by using a Zenon AlexFluor 647 labeling kit (Life Technologies). For CXCR4 staining, before fixation, the cells were incubated with Streptavidin-PE (BD Biosciences) for 30 min and washed. Apoptosis of human HSPCs was analyzed with Annexin V-PE (BD Biosciences) in accordance with the protocol of the manufacturer. To analyze the expression of Jagged-1 on murine stromal cells, femurs of NSG mice were dissected, crushed, and incubated in 0.1 % collagenase II in PBS (Sigma-Aldrich) in 37 °C for 90 min. Then the cell suspension was filtered via 70-μm mesh, washed in PBS, and stained with anti-mouse antibodies: anti-Jagged-1 PE antibody (eBioscience, San Diego, CA, USA), anti-CD31 and anti-rat IgG BV421 (BioLegend), and anti-CD45 FITC (BD Biosciences). At least 400 (450–700) CD45^−^CD31^+^ cells were collected in a flow cytometer. Phosphorylation of STAT3 in human HSPCs was assessed by intracellular staining with anti-pSTAT3-PE (pY705) antibody after permabilization with Phosphoflow Perm Buffer II (all from BD Biosciences) in accordance with the instructions of the manufacturer. Samples were analyzed by using a FACS Canto II cytometer (BD Biosciences) with Diva software. Approximately 100,000 cells were collected for analysis. Data analysis was performed with FlowJo software (Tree Star, Inc., now part of FlowJo LLC, Ashland, OR, USA). ModFit software was applied to analyze proliferation of in vitro cultured BM CD34^+^ cells. All used anti-human antibodies were tested for cross-reactivity with cells of non-transplanted mice, and no staining was present.

### In vivo inhibition of Notch signaling

To inhibit the Notch signaling pathway, mice were treated with DAPT, a γ-secretase inhibitor. Two hours before CLP surgery, hu-mice received i.p. injection of DAPT (Sigma-Aldrich) (60 mg/kg) dissolved in 50 μl of DMSO. The effects of Notch inhibition on human HSPCs were evaluated 24 h after CLP.

### Colony-forming unit assay

After erythrocyte lysis with Red Blood Cell Lysing Buffer (Sigma-Aldrich), BM cells (5 × 10^5^) from humanized mice were used for the hematopoietic colony-forming unit (CFU) assay. The human methylcellulose-based medium (R&D Systems) contains 25 % FBS, 2 % bovine serum albumin, 2 mM L-glutamine, and 5×10^-5^ M of mercaptoethanol and is supplemented with rh-SCF (50 ng/ml), rh-GM-CSF (10 ng/ml), rh-IL-3 (10 ng/ml), and rh-Epo (3 IU/ml). Cells were plated in duplicate in 35-mm Petri dishes. After 20 days of culture in a humidified incubator in 37 °C and 5 % CO_2_, the colonies were counted under an inverted microscope. CFU-GM (granulocyte macrophage), CFU-G (granulocyte), CFU-M (macrophage), and CFU-E (erythroid)/burst-forming unit-erythroid (BFU-E) colonies were identified. The medium was tested for supporting growth of murine BM cells prior to experiments, and no colonies were formed by cells from non-transplanted mice.

### Statistical analysis

Results are expressed as mean value ± standard deviation. Absolute numbers of particular cell populations were calculated from the total cell count and frequency of given population. Statistical comparison between the matched control and experimental animals was performed with non-parametric Wilcoxon paired test. For multiple comparisons, the one-way analysis-of-variance test with Tukey’s post test was used after testing the normal distribution of data sets with Kolmogorov-Smirnov test. A *P* value of less than 0.05 was considered significant. Each group consisted of three to eight mice. GraphPad Prism 5 software (GraphPad Software, Inc., San Diego, CA, USA) was used for all statistical analyses.

## Results

### Development of the human multilineage reconstitution in NSG mice

To enable a robust engraftment of human cells in the NSG recipients, we performed a conditioning myeloablation with busulfan as reported by others [22]. The applied regimen was well tolerated by all animals. Xenotransplantation of 10^5^ purified UCB CD34^+^ cells after 8 weeks resulted in the BM engraftment by human cells at an average of 57.5 ± 22 %. This percentage is higher than reported previously [22], but the increase can be attributed to the modified regimen of the busulfan treatment (2 × 25 mg/kg) that we used.

**Fig. 1:**
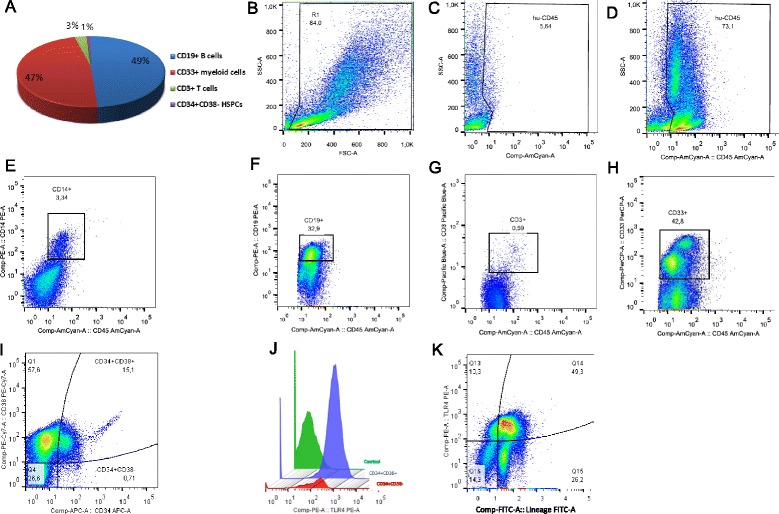
Development of human hematopoietic cells in the bone marrow of NSG mice 8 weeks after transplantation of human CD34^+^ cells. Representative dot plots show flow cytometry analysis of bone marrow cells with specific anti-human monoclonal antibodies. **a** Diagram showing mean frequency of human leukocyte subsets in the bone marrow of humanized mice. **b** Staining of bone marrow from non-humanized NSG mouse with anti-human CD45. **c** Morphology of bone marrow cells from hu-NSG. **d** Expression of pan-hematopoietic CD45 antigen in cells gated in R1. **e** Analysis of monocytes. **f** Analysis of B cells. **g** Analysis of T cells. **h** Analysis of myeloid cells. **i** Analysis of hematopoietic stem and progenitor cells (gated from R1). **j** Histogram comparing expression of TLR4 receptor on HSCs and progenitor cells (gated from i). **k** Expression of TLR4 is present on both undifferentiated Lineage^−^ cells and mature Lineage^+^ cells. The values on graphs present percentages of a given population from a maternal gate. *HSC* hematopoietic stem cell, *hu-NSG* humanized NOD.Cg-Prkdc/scidIL2rγ, *NSG* NOD.Cg-Prkdc/scidIL2rγ, *TLR* Toll-like receptor

Among all human hematopoietic cells, we identified HSPCs as well as differentiated myeloid and lymphoid cells (Fig. 1). Although human erythrocytes were not detected in the peripheral blood, the BM contained cells from the erythroid lineage as determined by the expression of hu-glycophorin A (CD235a). In the peripheral blood, the human CD45^+^ leukocytes were present at frequencies of 1–29 %, mostly being CD19^+^ or CD33^+^ (data not shown). Within the splenocytes, hu-CD45^+^ cells were present at the average of 17 ± 9 %. Human hematopoietic cells residing in the murine BM expressed TLR4: the antigen was present on both differentiated (Lineage^+^) and the Lineage^−^ undifferentiated cells (Fig. 1j). Both CD34^+^ CD38^−^ HSCs and CD34^+^ CD38^+^ progenitors (Fig. 1h) expressed TLR4; the TLR4 surface density was higher on the latter cells (Fig. 1i).

In summary, a substantial difference in the frequency of particular leukocyte populations in hu-NSG mice and humans exists. However, a high frequency of hu-myeloid cells, together with expression of hu-TLR4 receptor, makes those mice a suitable model for studying human-specific immune-inflammatory responses elicited by sepsis and endotoxemia.

### Endotoxemia and CLP modulated human hematopoietic stem and progenitor cell populations in humanized mice

All animals survived the administered dose of LPS, and the mortality in the CLP model reached 40 % after 24 h. As specified in the Methods section, mice were matched for experimental pairs on the basis of the frequency of human chimerism in the peripheral blood of mice analyzed 7 days before experiments. The pairing was efficient as the correlation coefficients (r) between groups were 0.899 (*P* = 0.016 in CLP experiments) and 0.677 (*P* = 0.022 in LPS experiments).

**Fig. 2:**
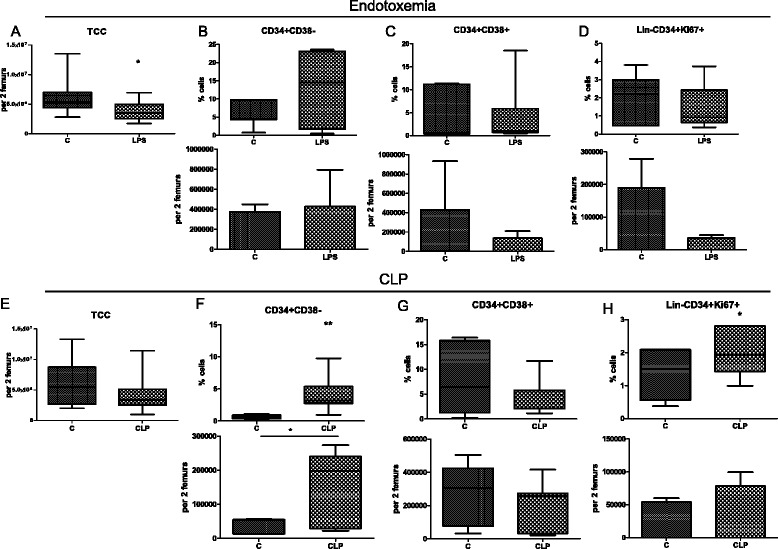
Analysis of human HSPC subpopulations in the murine bone marrow after induction of experimental sepsis. Eight weeks after transplantation of human umbilical cord blood CD34^+^ cells, mice were subjected to endotoxemia (40 μg of LPS intravenous) or cecal ligation and puncture surgery (CLP). Twenty-four hours later, bone marrow cells were harvested, counted, and stained with anti-human antibodies for flow cytometry analysis. Graphs in the *upper panel* (**a**, **b**, **c**, **d**) present results obtained in the model of endotoxemia, and graphs in the *lower panel* (**e**, **f**, **g**, **h**) impact of CLP on the human HSCPs. n = 6, *P < 0.05, **P < 0.01. *C* control, *HSPC* hematopoietic stem and progenitor cell, *LPS* lipopolysaccharide, *TCC* total cell count

After 24 h, cellularity of the BM was markedly reduced after both endotoxemia (3.7 × 10^6^ ± 1.4 × 10^6^/ml versus 5.9 × 10^6^ ± 2.7 × 10^6^/ml, *P* < 0.05) and CLP (3.6 × 10^6^ ± 1.6 × 10^6^ versus 6.3 × 10^6^ ± 3.7 × 10^6^/ml, *P* > 0.05, Fig. 2a, e). CLP-induced sepsis increased both the frequency (3.8 ± 1.1 % versus 0.65 ± 0.32 %, *P* < 0.01, Fig. 2f) and total cell count (1.5 × 105 ± 0.8 × 10^5^/ml versus 0.3 × 10^5^ ± 0.2 × 10^5^/ml, *P* < 0.05, Fig. 2f) of the early CD34^+^ CD38^−^ HSPCs. However, the frequency of the more committed CD34^+^ CD38^+^ progenitors did not change significantly after CLP. After LPS injection, the number of CD34^+^ CD38^−^ cells was increased, but the difference did not reach significance (13 ± 9.6 % versus 5.4 ± 2.9 %, *P* > 0.05, Fig. 2b).

We also analyzed the proliferative status of the human HSPCs by staining Ki-67, a protein indicative of cells that are in the active phases of the cell cycle. The frequency of Lin^−^CD34^+^ Ki-67^+^ HSPCs was significantly increased 24 h after induction of CLP (2.08 ± 0.68 % versus 1.36 ± 0.69 %) but not after endotoxemic challenge (Fig. 2d, h). Both models induced expansion of early HSPCs in the BM, and the effect on the progenitor cells was insignificant. Finally, we assessed the more differentiated population of human cells; CLP-induced sepsis led to a 3.5-fold reduction of CD33^+^ myeloid cells (16.98 % versus 4.89 %, *P* < 0.05), but the impact of sepsis on CD20^+^ B cells was insignificant.

### Endotoxemia and CLP reduced hematopoietic colony forming by human cells

**Fig. 3:**
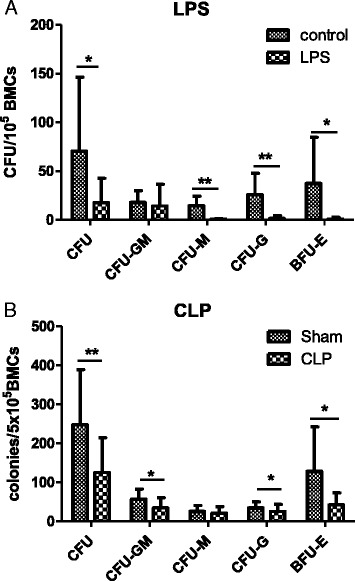
Analysis of the growth of human hematopoietic colonies from bone marrow cells isolated from humanized mice 24 h after induction of experimental sepsis. Twenty-four hours after induction of experimental sepsis, bone marrow cells (5 × 10^5^) from hu-NSG were seeded on methylocellulose medium supplemented with human growth factors. After 20 days of culture, the colonies formed by human progenitor cells were counted. **a** Results from the model of endotoxemia. **b** Results from the CLP-induced sepsis model. n = 6, *P < 0.05, **P < 0.01. *BMC* bone marrow cell, *CLP* cecal ligation and puncture surgery, *hu-NSG* humanized NOD.Cg-Prkdc/scidIL2rγ

In the next step, we aimed to test the impact of endotoxemia and CLP on the functionality and number of hu-hematopoietic progenitors. To do this, we used the CFU assay with medium supplemented with recombinant human cytokines which do not support growth of murine cells. The differences in baseline production of CFUs from control mice in both models (Fig. 3) are a result of different human chimerism after transplantation, but each time control mice were paired with experimental mice on the basis of human cell chimerism. Human hematopoietic cells isolated from the BM of mice injected with LPS formed four-fold fewer colonies compared with control mice. A similar effect occurred in cells from CLP mice: a two-fold reduction was observed.

Specifically, endotoxemia significantly reduced the growth of CFU-G (2 ± 2 versus 26 ± 20) and CFU-M (0.85 ± 0.5 versus 14.5 ± 9). In CLP mice, this phenomenon was less dramatic in CFU-G (25.5 ± 17 versus 35 ± 14 in control, *P* < 0.05), but the number of CFU-M was not affected (21.5 ± 15 versus 25.9 ± 13.9). In contrast, CLP reduced the CFU-GM formation by 39 % (35 ± 23 versus 57 ± 23 in control, *P* < 0.05), but endotoxemia did not influence that type of progeny. Both models induced a markedly decreased the growth of BFU-E: in endotoxemia by 37-fold and in CLP by three-fold (Fig. 3). In summary, both models significantly diminished the functional activity of human HPCs from the humanized BM.

### Endotoxemia and CLP upregulated expression of TLR4 and CXCR4 on human HSPCs

**Fig. 4:**
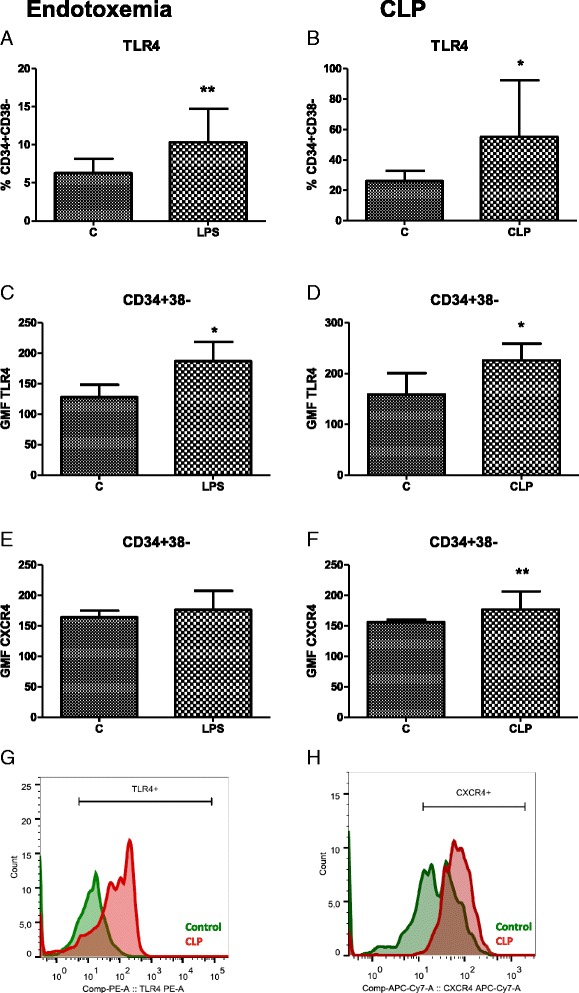
Modulation of the expression of TLR4 and CXCR4 receptors on human HSCs. Changes of TLR4 (**a**, **c**) and CXCR4 (**e**) expression on CD34^+^ CD38^−^ cells 24 h after single LPS injection. Impact of CLP model on the modulation of TLR4 receptor expression (**b**, **d**) and CXCR4 expression (**f**). Representative histograms of TLR4 (**g**) and CXCR4 (**h**) expression on CD34^+^ CD38^−^ HSCs from control and CLP mice are presented. n = 6, *P < 0.05, **P < 0.01. *CLP* cecum ligation and puncture, *GMF* geometric mean fluorescence, *HSC* hematopoietic stem cell, *LPS* lipopolysaccharide, *TLR* Toll-like receptor

Given that the HSPC fate can be influenced via TLR receptors and that TLR4-mediated recognition of pathogens is considered a key signaling axis in Gram-negative and polymicrobial infections, we then analyzed modulation of the TLR4 expression. In CLP sepsis, there was a two-fold increase in the frequency of the TLR4-positive CD34^+^ CD38^−^ cells (55 ± 13.4 % versus 26.1 ± 13.4 %; Fig. 4a-d). Moreover, the geometric mean fluorescence of anti-TLR4 staining increased by 43 % (Fig. 4b, d, g). Similar results were obtained in endotoxemia: the TLR4-positive CD34^+^ CD38^−^ cells and the fluorescence intensity increased by 63 % and 46 %, respectively (Fig. 4a, c).

Because the retention and mobilization of HSPCs can contribute to changes in their BM quantity, we also attempted to evaluate the impact of sepsis on the expression of CXCR4, the key receptor involved in the maintenance of HSCs in the BM. CLP increased the density of CXCR4 on CD34^+^ CD38^−^ cells (Fig. 4e, h), whereas LPS had no effect on CXCR4 (Fig. 4f).

### LPS stimulated proliferation and differentiation of primary human BM CD34^+^ cells in vitro

**Fig. 5:**
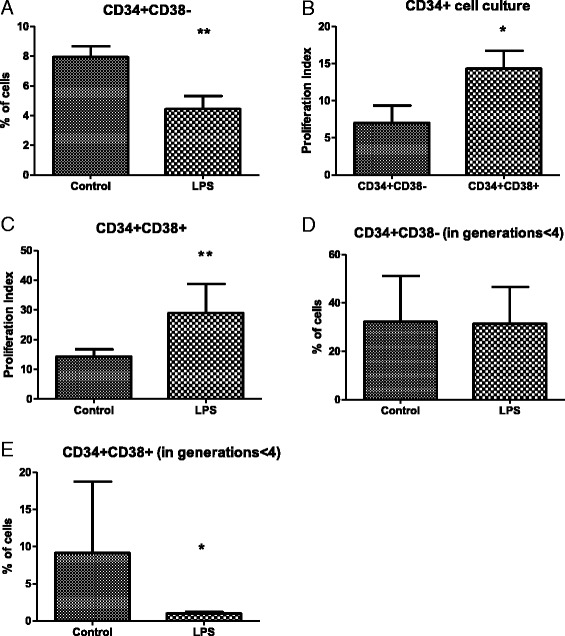
Impact of LPS on the differentiation and proliferation of purified CD34^+^ human BM cells in vitro. **a** Effect of LPS on the frequency of the CD34^+^ CD38^−^ subpopulation after 9 days of culture. **b** Effect of LPS on the proliferative index of CD34^+^ CD38^+^ subpopulation. **c** Proliferation of CD34^+^ CD38^+^ cells in the presence of LPS. Impact of LPS on the frequency of CD34^+^ CD38^−^ cells (**d**) and CD34^+^ CD38^+^ cells (**e**) within first, second, and third generations of daughter cells (fewer than four generations). All results obtained after 9 days of culture in atmosphere of 1 % O_2_. n = 6, *P < 0.05, **P < 0.001. *BM* bone marrow, *LPS* lipopolysaccharide

The modulation of hu-HSCs in in vivo models is the sum of complex signaling stimuli triggered by sepsis. Thus, we investigated how LPS-induced TLR4 stimulation influences proliferation of purified HSPCs. After 9 days of culture, the presence of LPS reduced the frequency of CD34^+^ CD38^−^ HSCs by 44 % compared with control (Fig. 5a). As the cells were stained with a fluorescent dye (CellTrace Violet), we were able to trace the proliferation history of each cell. When cultured in the medium alone, CD34^+^ CD38^+^ cells displayed higher proliferative activity (defined by proliferative index [23]) in comparison with CD34^+^ CD38^−^ cells (14.3 ± 5.4 versus 7 ± 2.1, *P* < 0.05) (Fig. 5b). The addition of LPS did not influence the proliferative index of CD34^+^ CD38^−^ HSCs, but the index strongly increased in CD34^+^ CD38^+^ cells (*P* < 0.001) (Fig. 5c). We also calculated frequency of the daughter cells in the first, second, and third generations. LPS did not change the frequency of CD34^+^ CD38^−^ cells within the sum of fewer than four generations, but it significantly decreased frequency of the CD34^+^ CD38^+^ cells in fewer than four generations (by nine-fold) (Fig. 5d, e). These observations suggest that LPS accelerated the proliferation of the more differentiated CD34^+^ CD38^+^ cell population.

### Endotoxemia modulated the Jagged-1 expression in the bone marrow

**Fig. 6:**
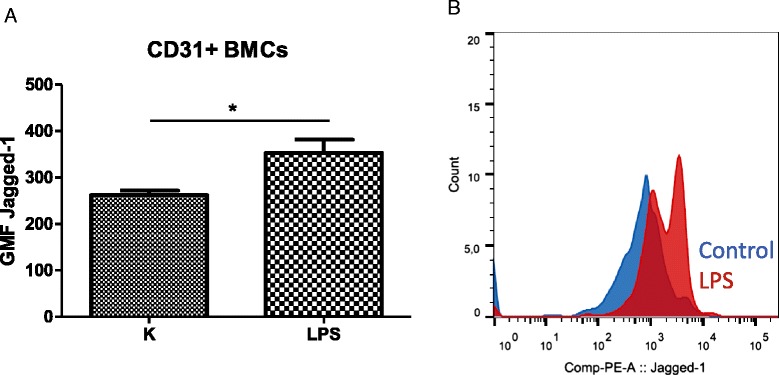
Endotoxemia modulates expression of Jagged-1, a Notch-1 ligand on the bone marrow endothelial cells. Twelve hours after injection of LPS (40 μg), murine femurs were crushed and digested with collagenase II, and then cells were stained with anti-mouse CD45, anti-mouse CD31, and anti-mouse Jagged-1 antibodies. Expression of Jagged-1 on murine CD45^−^CD31^+^ endothelial cells was measured (**a**). **b** Representative histograms overlay of the expression of Jagged-1 after LPS injection and in control mice. n = 6, *P < 0.05. *BMC* bone marrow cell, *GMF* geometric mean fluorescence, *LPS* lipopolysaccharide

The Notch signaling pathway is co-responsible for maintaining the quiescent HSCs. Therefore, in the last step, we evaluated the expression of Notch-1 ligand Jagged-1 in the murine marrow stromal cells. Twelve hours after the LPS injection, the expression of Jagged-1 was upregulated on the CD45^−^ fraction and CD45^−^CD31^+^ endothelial cells by 35 % (*P* < 0.05) (Fig. 6).

### Sepsis activated the Notch signaling pathway that was responsible for the expansion of human HSCs

To assess the contribution of Notch signaling pathway in the expansion of human HSCs during CLP sepsis, we treated hu-mice with DAPT prior to the CLP surgery. DAPT is a γ-secretase inhibitor known to efficiently block the cleavage of the NICD which subsequently activates transcription of downstream target genes [24]. The activation of Notch-1 signaling was detected by staining of its cytoplasmic subunit, NICD.

**Fig. 7:**
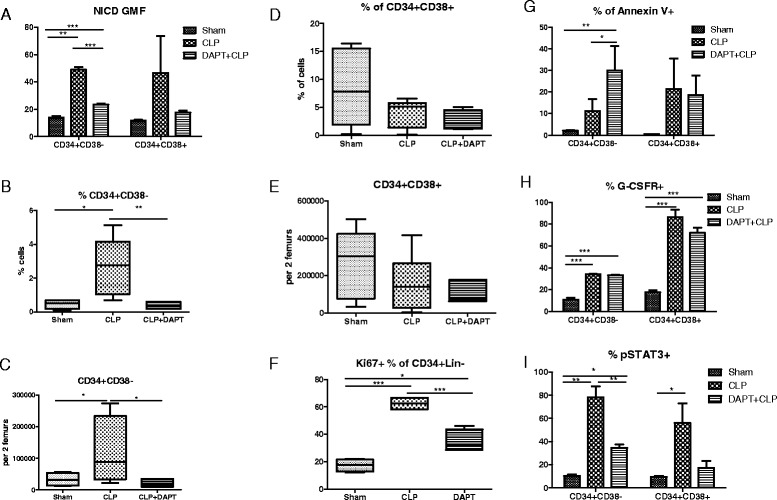
Effects of Notch pathway inhibition in human HSPCs 24 h after induction of CLP sepsis in hu-mice. Pretreatment with DAPT (60 mg/kg) 2 h before CLP surgery inhibited activation of Notch-1 assessed by fluorescence intensity of NICD (**a**). Impact of the inhibition of Notch signaling on the CLP-induced expansion of CD34^+^ CD38^−^ HSCs (**b**, **c**) and effect on CD34^+^ CD38^+^ progenitors (**d**, **e**). Influence of DAPT treatment on the frequency of proliferating HSCs (**f**), and the rate of apoptosis of CD34^+^ CD38^−^ cells (**g**). Effect of DAPT on the expression of G-CSFR on HSPCs after CLP (**h**), and the phosphorylation of STAT3 (**i**). n = 3–5. *P < 0.05, **P < 0.001, ***P < 0.001. *CLP* cecum ligation and puncture, *G-CSFR* granulocyte colony-stimulating factor receptor, *GMF* geometric mean fluorescence, *HSC* hematopoietic stem cell, *HSPC* hematopoietic stem and progenitor cell, *NICD* Notch-1 intracellular domain

CLP led to a significant increase in the fluorescence of NICD in CD34^+^ CD38^−^ cells which was abrogated by DAPT pretreatment (Fig. 7a). This effect was similar in CD34^+^ CD38^+^ cells, although the differences did not reach significance (Fig. 7a). The Notch blockade resulted in a seven-fold reduction of the expansion of primitive CD34^+^ CD38^−^ (Fig. 7b, c) but had no effect on the more mature CD34^+^ CD38^+^ population (Fig. 7d, e). In addition, the percentage of actively proliferating CD34^+^ Lin^−^ HSCs decreased in the presence of DAPT from 58 % to 37 % (*P* < 0.001, Fig. 7f).

To analyze the mechanisms controlling the HSC number, the rate of apoptosis was also measured. CLP alone increased the rate of Annexin V-positive CD34^+^ CD38^−^ HSCs by five-fold (*P* > 0.05), and DAPT treatment further augmented this rate by 15-fold compared with sham animals (*P* < 0.001, Fig. 7g).

As the response to bacterial infections often evokes the G-CSF-driven process of emergency myelopoiesis [4], we analyzed the impact of sepsis on the expression of G-CSF receptors on human HSPCs. CLP increased the expression of G-CSFR on the surface of CD34^+^ CD38^−^ cells (*P* < 0.05) and CD34^+^ CD38^+^ cells (*P* > 0.05) and this effect was independent of the Notch activity (Fig. 7h).

The G-CSFR-induced stimulation of HPCs during emergency hematopoiesis is mediated by phosphorylation of STAT3 protein which activates translation of downstream genes [4]. Sepsis increased the percentages of both CD34^+^ CD38^−^ and CD34^+^ CD38^+^ with phosphorylated STAT3 and this effect was diminished by DAPT treatment, suggesting partial crosstalk between these two pathways (Fig. 7i). These results imply broad effects of Notch signaling in the proliferation and survival of HSCs during sepsis.

## Discussion

This is the first study in which humanized mice were employed to study human HSPCs in the BM niche during experimental sepsis and endotoxemia. Both challenges in the chimeric mice provoked an active expansion of the primitive HSC populations with a concomitant decrease of the more committed progenitor pool. Moreover, we show that the changes observed in humanized mice were in contrast to those recorded after in vitro stimulation of HSPCs with LPS. Those findings underscore the importance of complexity of signal interactions in the BM and advance knowledge on the immune system reprogramming occurring during severe sepsis.

Similarly to other studies, the main population of the hu-BM cells consisted of B cells [25], whereas T cells constituted only 3 % of the total BM cells. The recapitulation of human sepsis T-cell response in hu-mice has already been reported by the Hotchkiss group [16]. In their study, the CLP sepsis induced apoptosis of T cells and reduced the delayed-type hypersensitivity response [16]. Also, the myeloid cells in hu-mice were reported to display functional responses to cytokine stimulation (phosphorylation of STAT3 and STAT5 in response to G-CSF and STAT1, STAT3, and STAT5 after interferon-gamma treatment) [26].

Given that our mice developed a substantial percentage of CD33^+^ cells, they appear adequately competent to recapitulate such a response to sepsis. Human cells in the BM of our hu-mice express the TLR4 receptor, the major pattern-recognition receptor for LPS. TLR4 receptor was present on both differentiated cells (Lineage^+^) and HSPCs (Fig. 1h, i). Interestingly, 8 weeks after transplantation, hu-CD34^+^ cells constituted up to 13 % of human BM cells, and the CD34^+^ CD38^−^ subpopulation was up to 5 %. These values are significantly higher than in the human BM, where those cell populations constitute, on average, 1.4 % and 0.1 %, respectively [27]. This discrepancy can be due to either an increased capacity for self-renewal of the hu-HSPCs or their differentiation impairment in the murine BM niche. Altogether, our current model is suitable to study the impact of sepsis on human HSPCs.

To provide a wide coverage to the studied aim, we employed two different models, namely non-microbial endotoxemia and polymicrobial CLP. Because they differ in the kinetics of cytokine response [28] and in several other discrepancies existing between those two models [29], this enabled us to investigate potential differences in HSPC responses in two highly inflammatory milieus of different origin.

The uniform reduction in the BM cellularity after endotoxemia and sepsis can be explained by mobilization of myeloid cells to the peripheral blood and spleen [30]. However, the same number of BM cells from septic animals gave a lower number of hematopoietic colonies. This was observed in all types of colonies and in both models yet with a stronger suppression of CFU-M and CFU-G in endotoxemia. Interpretation of our findings is difficult as no similar studies have been performed in humanized mice to date; we can only compare our results with those obtained in immunocompetent (non-humanized) mice. Rodriguez et al. reported a similar inhibition of myeloid colony formation after injection of LPS from *P. aeruginosa* in C57/BL6 mice [12]. Also, a decrease of the erythroid colony formation was shown after induction of peritoneal sepsis in vivo as well as in vitro LPS stimulation of the BM cells [31]. In contrast, in different pre-clinical models, sepsis exerted a stimulatory effect on myeloid colonies [31, 32]. The discrepancy of the myeloid progenitor activity between the abovementioned studies may be attributed to differences between the models themselves or their severity or both. In the future, it would be of interest to compare dynamic changes in the HSPC compartment of humanized mice subjected to sepsis of different severity.

Both endotoxemia and sepsis increased the frequency and the total cell count of CD34^+^ CD38^−^ cells in the BM of hu-NSG. Especially, the increase of the total number of those cells indicates that their expansion progressed in an active manner. Support for this claim comes from the upregulation in the expression of the Ki-67 protein that is expressed in cycling cells [33]. Although CLP had a much stronger effect on the expression of Ki-67 in HSPCs, endotoxemia also increased frequency of proliferating cells. More differentiated CD34^+^ CD38^+^ cells were not significantly affected in our models, but a tendency to their reduction could be seen after LPS injection. CLP also reduced the pool of BM human CD33^+^ myeloid cells similarly to wild-type models [30]. As it was shown that the mobilization of granulocytes from BM ‘paves the way’ for egress of progenitor cells [34], this mechanism can contribute to the decrease of the BM progenitor pool in our model. Our results are consistent with data from a different type of endotoxemic insult: an infusion of *P. aeruginosa* LPS increased early HSCs but inhibited the colony formation [12].

The role of TLR4 signaling in HSPCs during infections has been controversial. On one hand, the expansion of HSCs from *TLR4*^−/−^ mice to LPS was absent [11, 12]; on the other hand, those mice showed unchanged response to CLP and peritonitis [11, 35]. Nevertheless, recent reports prove the importance of TLR4 signaling in the steady-state granulopoiesis [36]. Interestingly, in our models, the expression of TLR4 was upregulated on both CD34^+^ CD38^−^ and CD34^+^ CD38^+^ cells and this is in contrast to modulation of those receptors on monocytes from patients with sepsis [37]. This collectively suggests an active participation of TLR4 in the response of HSPCs to sepsis. Moreover, in the CLP sepsis model, expression of CXCR4 on HSPCs was increased. A similar LPS-induced upregulation was observed in neutrophils, B cells, and murine HSPCs [38–40]. Such a receptor modulation may be responsible for enhanced retention of HSPCs in the BM or peripheral tissues [41].

To unravel the role of the BM microenvironment in the sepsis-induced HSC expansion, we traced the proliferation history of purified hu-CD34^+^ BM cells after culturing them in vitro with LPS. The results of the LPS experiment are in a stark contrast to the results obtained in hu-mice: LPS decreased the pool of CD34^+^ CD38^−^ cells and strongly stimulated divisions of differentiated CD34^+^ CD38^+^ cells. We interpret this in vitro-versus-in vivo discrepancy as proof for a strong influence of the BM microenvironment. To date, several important components of the BM niche have been recognized. As we maintained the cell culture in a hypoxic atmosphere that resembles conditions of the BM niche [42], we doubt that this factor had influenced the observed differences. Although we stimulated purified CD34^+^ cells with purified LPS, it was shown that CD34^+^ HSPCs are potent producers of cytokines and growth factors [43] and that the TLR4 signaling even increases production of cytokines by HSPCs (verified for murine cells [7]). Thus, cytokines produced in such an autocrine manner were likely present in our 9-day culture similarly to the BM milieu.

Altogether, we hypothesize that reshaping of the intercellular signaling in the BM niche may be responsible for the expansion and blocking of HSC differentiation in hu-mice. To identify a potential candidate responsible for this phenomenon, we evaluated the Jagged-1 (a ligand for Notch-1 receptor) expression after endotoxemia. Notch-1 signaling is a well-known, evolutionally conserved pathway that stimulates expansion and inhibits differentiation of early HSCs [44]. Indeed, LPS upregulated Jagged-1 expression on marrow stromal and endothelial cells. Then, we confirmed that CLP sepsis activated Notch-1 signaling in HSPCs, demonstrating that this pathway was responsible for the sepsis-induced proliferation and expansion of HSCs. Furthermore, this proliferation was dependent, at least partially, on the activation of STAT3. Concomitant upregulation of G-CSFR on HSCs during sepsis, a known inducer of STAT3 phosphorylation during emergency myelopoiesis, is probably responsible for the majority of STAT3 activation [45]. The crosstalk between Notch and STAT3 pathways was reported before [46], and our results suggest that such an interaction may be important during severe infections in HSCs. Interestingly, sepsis itself induced apoptosis (annexin V binding) in HSCs and to a greater extent in HPCs. Inhibition of Notch signaling by DAPT further increased the rate of apoptosis of HSCs but not HPCs. This finding underlines the exclusive importance of the Notch pathway in maintaining the expansion of more primitive HSCs. Therefore, it may be hypothesized that the activation of Notch in HSCs during sepsis serves as a mechanism that protects those cells from exhaustion during massive infections but may also reduce their maturation into more differentiated progenitors.

Our study has several drawbacks. Although hu-mice develop human immunocompetent cells, their composition does not ideally reflect the physiological human immune system. Moreover, the human cells are not fully differentiated. Thus, particular defense and regulatory mechanisms may be partially impaired or deregulated or both. Another limitation is the partial cross-reactivity of murine cytokines with human cells (e.g., M-CSF and interleukin-15 [47]).

## Conclusions

In summary, we report that both endotoxemia and CLP induced proliferation and accumulation of human HSPCs in the BM niches but that the pool of hematopoietic progenitors strongly decreased. As this phenomenon was not recapitulated in the in vitro LPS stimulation of purified HSPCs, it highlights the importance of the BM microenvironment in modulation of HSPCs during sepsis. Furthermore, our findings suggest that increased Notch-1 signaling via Jagged-1 contributed to the abovementioned modulation. It is suggested that the impairment of HSC maturation in endotoxemia and sepsis may have significant implications in the development and progress of various immune disturbances in affected patients.

## Abbreviations

BFU-E: Burst-forming unit-erythroid
BM: Bone marrow
CFU: Colony-forming unit
CFU-G: Colony-forming unit-granulocyte
CFU-GM: Colony-forming unit-granulocyte macrophage
CFU-M: Colony-forming unit-macrophage
CLP: Cecum ligation and puncture
DMSO: Dimethyl sulfoxide
FBS: Fetal bovine serum
G-CSF: granulocyte colony-stimulating factor
G-CSFR: granulocyte colony-stimulating factor receptor
HPC: Hematopoietic progenitor cell
HSC: Hematopoietic stem cell
HSPC: Hematopoietic stem and progenitor cell
hu-NSG: Humanized NOD.Cg-Prkdc/scidIL2rγ
i.p.: Intraperitoneal
LPS: Lipopolysaccharide
LT-HSC: long-term hematopoietic stem cell
NICD: Notch Intracellular Domain
NSG: NOD.Cg-Prkdc/scidIL2rγ
PBS: Phosphate-buffered saline
TLR: Toll-like receptor
UCB: Umbilical cord blood
